# Supplementary material for: Causal Relationship between Aging and Anorexia Nervosa: A White-Matter-Microstructure-Mediated Mendelian Randomization Analysis
Source: Biomedicines. 2024 Aug 16;12(8):1874. doi: 10.3390/biomedicines12081874 (PMC11351342; doi:10.3390/biomedicines12081874)
Supplement: Supplementary file 1 [file biomedicines-12-01874-s001.zip › Supplementary Figure/Figure S4/Singe SNP plot of MR analysis for TL to ubm-b-1571.pdf]

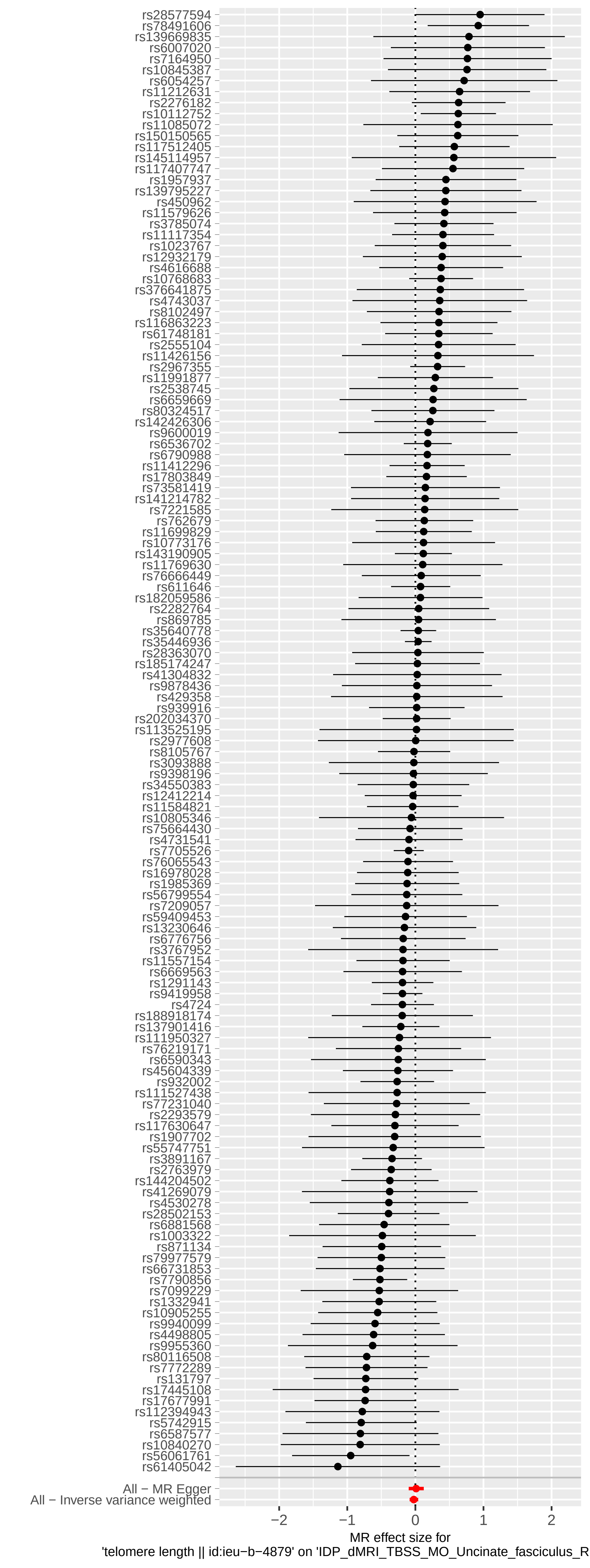
rs28577594
rs78491606
rs139669835
rs6007020
rs7164950
rs10845387
rs6054257
rs11212631
rs2276182
rs10112752
rs11085072
rs150150565
rs117512405
rs145114957
rs117407747
rs1957937
rs139795227
rs450962
rs11579626
rs3785074
rs11117354
rs1023767
rs12932179
rs4616688
rs10768683
rs376641875
rs4743037
rs8102497
rs116863223
rs61748181
rs2555104
rs11426156
rs2967355
rs11991877
rs2538745
rs6659669
rs80324517
rs142426306
rs9600019
rs6536702
rs6790988
rs11412296
rs17803849
rs73581419
rs141214782
rs7221585
rs762679
rs11699829
rs10773176
rs143190905
rs11769630
rs76666449
rs611646
rs182059586
rs2282764
rs869785
rs35640778
rs35446936
rs28363070
rs185174247
rs41304832
rs9878436
rs429358
rs939916
rs202034370
rs113525195
rs2977608
rs8105767
rs3093888
rs9398196
rs34550383
rs12412214
rs11584821
rs10805346
rs75664430
rs4731541
rs7705526
rs76065543
rs16978028
rs1985369
rs56799554
rs7209057
rs59409453
rs13230646
rs6776756
rs3767952
rs11557154
rs6669563
rs1291143
rs9419958
rs4724
rs188918174
rs137901416
rs111950327
rs76219171
rs6590343
rs45604339
rs932002
rs111527438
rs77231040
rs2293579
rs117630647
rs1907702
rs55747751
rs3891167
rs2763979
rs144204502
rs41269079
rs4530278
rs28502153
rs6881568
rs1003322
rs871134
rs79977579
rs66731853
rs7790856
rs7099229
rs1332941
rs10905255
rs9940099
rs4498805
rs9955360
rs80116508
rs7772289
rs131797
rs17445108
rs17677991
rs112394943
rs5742915
rs6587577
rs10840270
rs56061761
rs61405042
All − MR Egger
All − Inverse variance weighted
−2
−1
0
1
2
MR effect size for
'telomere length || id:ieu−b−4879' on 'IDP_dMRI_TBSS_MO_Uncinate_fasciculus_R
